# Bis[tris­(4-fluoro­phen­yl)phosphine-κ*P*](tropolonato-κ^2^ *O*,*O*′)copper(I)

**DOI:** 10.1107/S1600536809010630

**Published:** 2009-03-28

**Authors:** Gideon Steyl

**Affiliations:** a Department of Chemistry, University of the Free State, Bloemfontein 9300, South Africa

## Abstract

The title compound, [Cu(C_7_H_5_O_2_)(C_18_H_12_F_3_P)_2_], a copper(I) tris­(4-fluoro­phen­yl)phosphine tropolonate derivative, is the first tropolonate complex with fluorinated aryl­phosphine ligands. The Cu^I^ atom has a distorted tetra­hedral coordination; the most important geometrical parameters of the mol­ecule are: Cu—P = 2.2377 (10) and 2.2335 (15) Å, Cu—O = 2.084 (2) and 2.082 (2) Å, O—Cu—O = 77.72 (10)°, P—Cu—P = 128.82 (4)° and O—C—C—O = −2.1 (5)°.

## Related literature

The title compound is structurally related to the flavonolato and nitro­sophenyl­hydroxy­laminato derivatives (Speier *et al.*, 1990 ▶; Charalambous *et al.*, 1984 ▶). For related diketonato complexes, see: Hill & Steyl (2008 ▶); Steyl & Roodt (2006 ▶); Steyl (2007 ▶); Steyl & Hill (2009 ▶). For general background, see: Roodt *et al.* (2003 ▶); Crous *et al.* (2005 ▶). For the discovery of tropolone and its derivatives, see: Dewar (1945 ▶).
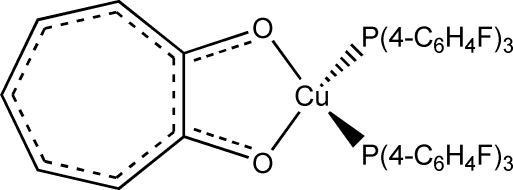


## Experimental

### 

#### Crystal data

[Cu(C_7_H_5_O_2_)(C_18_H_12_F_3_P)_2_]
*M* *_r_* = 817.14
Triclinic, 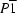

*a* = 10.570 (6) Å
*b* = 11.399 (1) Å
*c* = 15.861 (1) Å
α = 100.548 (6)°
β = 92.663 (5)°
γ = 98.677 (6)°
*V* = 1851.9 (11) Å^3^
*Z* = 2
Mo *K*α radiation
μ = 0.74 mm^−1^
*T* = 153 K
0.18 × 0.15 × 0.11 mm

#### Data collection

Bruker APEXII CCD diffractometer
Absorption correction: multi-scan (*SADABS*; Bruker, 1998 ▶) *T*_min_ = 0.878, *T*_max_ = 0.923
13924 measured reflections
7860 independent reflections
3579 reflections with *I* > 2σ(*I*)
*R*_int_ = 0.049

#### Refinement

*R*[*F* ^2^ > 2σ(*F* ^2^)] = 0.042
*wR*(*F* ^2^) = 0.103
*S* = 0.76
7860 reflections
487 parameters
H-atom parameters constrained
Δρ_max_ = 0.35 e Å^−3^
Δρ_min_ = −0.34 e Å^−3^

### 

Data collection: *APEX2* (Bruker, 2005 ▶); cell refinement: *SAINT-Plus* (Bruker, 2004 ▶); data reduction: *SAINT-Plus*; program(s) used to solve structure: *SHELXS97* (Sheldrick, 2008 ▶); program(s) used to refine structure: *SHELXL97* (Sheldrick, 2008 ▶); molecular graphics: *DIAMOND* (Brandenburg & Putz, 2006 ▶); software used to prepare material for publication: *SHELXL97*.

## Supplementary Material

Crystal structure: contains datablocks I, global. DOI: 10.1107/S1600536809010630/ya2084sup1.cif

Structure factors: contains datablocks I. DOI: 10.1107/S1600536809010630/ya2084Isup2.hkl

Additional supplementary materials:  crystallographic information; 3D view; checkCIF report

## Figures and Tables

**Fig. 1.**
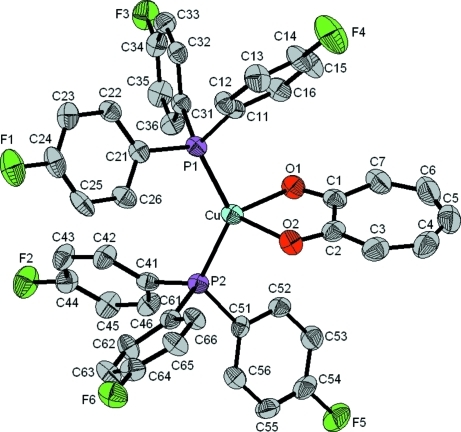
Molecular structure of the title compound, showing the numbering scheme and atomic displacement ellipsoids at the 50% probability level; hydrogen atoms are omitted for clarity.

## supplementary crystallographic information

### Comment

Tropolone and its derivatives have been of interest ever since their first discovery in the early 1940's (Dewar, 1945); they are known to have applications in pharmacology (Hill & Steyl, 2008) and catalysis (Crous *et al.*, 2005). Recently, reseach in this area has been extended to include phosphine metal complexes and the effect troplonato ligand has on the solid state and chemical behaviour of these complexes (Steyl, 2007; Steyl & Roodt, 2006; Roodt *et al.*, 2003). Only two other examples of copper triphenylphosphine complexes are known to date, which contain a five-membered O,O-bidentate chelating ring system, *i.e.*, the flavonolato and nitrosophenylhydroxylaminato derivatives (Speier *et al.*, 1990; Charalambous *et al.*, 1984). In this paper, the structure of tropolonato-bis[tri(4-fluorophenyl)phosphine]copper(I) complex is reported (Fig. 1).

The Cu—O and Cu—P bond distances are well within normal ranges; the bond angles at the Cu atom show significantly distorted tetrahedral coordination (Table 1). The bidentate bite angle O1—Cu—O2 77.72 (10)° is close to the analogous angles in the previously reported structures (Speier *et al.*, 1990; Charalambous *et al.*, 1984). The phosphine moieties adopt a staggered conformation, the C11—P1—Cu—O1 and C51—P2—Cu—O2 torsion angles being equal to -59.05 (14)° and -49.50 (14)°.

### Experimental

Sodium tropolonate (116 mg, 0.57 mmol) was added to dichloromethane solution (10 ml) of [Cu(P(p-C_6_H_4_F)_3_)_2_NO_3_] (396 mg, 0.57 mmol). On slow evaporation of the solvent, crystals suitable for X-Ray structural study were obtained. Yield: 300 mg (74%).

### Refinement

H atoms were positioned geometrically and refined using a riding model, with C—H = 0.95 Å and *U*_iso_(H) = 1.2*U*_eq_ of the carrier C atom.

### Crystal data

**Table:**

[Cu(C_7_H_5_O_2_)(C_18_H_12_F_3_P)_2_]	*Z* = 2
*M**_r_* = 817.14	*F*(000) = 832
Triclinic, *P*1	*D*_x_ = 1.465 Mg m^−^^3^
Hall symbol: -P 1	Mo *K*α radiation, λ = 0.71073 Å
*a* = 10.570 (6) Å	Cell parameters from 5934 reflections
*b* = 11.399 (1) Å	θ = 3.1–22.7°
*c* = 15.861 (1) Å	µ = 0.74 mm^−^^1^
α = 100.548 (6)°	*T* = 153 K
β = 92.663 (5)°	Cuboid, yellow
γ = 98.677 (6)°	0.18 × 0.15 × 0.11 mm
*V* = 1851.9 (11) Å^3^	

### Data collection

**Table:**

Bruker APEXII CCD diffractometer	7860 independent reflections
Radiation source: fine-focus sealed tube	3579 reflections with *I* > 2σ(*I*)
graphite	*R*_int_ = 0.049
Detector resolution: 512 x 512 pixels mm^-1^	θ_max_ = 27.0°, θ_min_ = 2.3°
φ and ω scans	*h* = −13→7
Absorption correction: multi-scan (*SADABS*; Bruker, 1998)	*k* = −14→14
*T*_min_ = 0.878, *T*_max_ = 0.923	*l* = −20→20
13924 measured reflections	

### Refinement

**Table:**

Refinement on *F*^2^	Primary atom site location: structure-invariant direct methods
Least-squares matrix: full	Secondary atom site location: difference Fourier map
*R*[*F*^2^ > 2σ(*F*^2^)] = 0.042	Hydrogen site location: inferred from neighbouring sites
*wR*(*F*^2^) = 0.103	H-atom parameters constrained
*S* = 0.76	*w* = 1/[σ^2^(*F*_o_^2^) + (0.0456*P*)^2^] where *P* = (*F*_o_^2^ + 2*F*_c_^2^)/3
7860 reflections	(Δ/σ)_max_ = 0.001
487 parameters	Δρ_max_ = 0.35 e Å^−^^3^
0 restraints	Δρ_min_ = −0.34 e Å^−^^3^

### Special details

**Table:**

Geometry. All e.s.d.'s (except the e.s.d. in the dihedral angle between two l.s. planes) are estimated using the full covariance matrix. The cell e.s.d.'s are taken into account individually in the estimation of e.s.d.'s in distances, angles and torsion angles; correlations between e.s.d.'s in cell parameters are only used when they are defined by crystal symmetry. An approximate (isotropic) treatment of cell e.s.d.'s is used for estimating e.s.d.'s involving l.s. planes.
Refinement. Refinement of *F*^2^ against ALL reflections. The weighted *R*-factor *wR* and goodness of fit *S* are based on *F*^2^, conventional *R*-factors *R* are based on *F*, with *F* set to zero for negative *F*^2^. The threshold expression of *F*^2^ > σ(*F*^2^) is used only for calculating *R*-factors(gt) *etc*. and is not relevant to the choice of reflections for refinement. *R*-factors based on *F*^2^ are statistically about twice as large as those based on *F*, and *R*- factors based on ALL data will be even larger.

### Fractional atomic coordinates and isotropic or equivalent isotropic displacement parameters (Å^2^)

**Table:**

	*x*	*y*	*z*	*U*_iso_*/*U*_eq_	
Cu	0.56504 (4)	0.64636 (4)	0.25757 (3)	0.03356 (14)	
P2	0.46439 (8)	0.46425 (8)	0.27118 (6)	0.0303 (2)	
P1	0.77346 (8)	0.70617 (8)	0.24545 (6)	0.0307 (2)	
F2	0.67563 (19)	0.1787 (2)	0.50523 (14)	0.0597 (6)	
F6	0.4430 (2)	0.12081 (19)	−0.06083 (13)	0.0562 (6)	
F3	1.0479 (2)	0.8577 (2)	0.59293 (13)	0.0562 (6)	
F5	−0.06950 (19)	0.4603 (2)	0.36866 (17)	0.0719 (8)	
F1	1.0658 (2)	0.3574 (2)	0.04772 (15)	0.0702 (7)	
O1	0.5103 (2)	0.7986 (2)	0.33161 (15)	0.0391 (6)	
O2	0.4337 (2)	0.6937 (2)	0.17298 (15)	0.0411 (6)	
F4	0.8496 (3)	1.1318 (2)	0.07640 (18)	0.0946 (9)	
C41	0.5270 (3)	0.3830 (3)	0.3488 (2)	0.0311 (8)	
C21	0.8670 (3)	0.6002 (3)	0.1862 (2)	0.0327 (9)	
C44	0.6268 (3)	0.2484 (3)	0.4544 (2)	0.0391 (9)	
C36	0.8146 (3)	0.7076 (3)	0.4198 (2)	0.0373 (9)	
H36	0.7380	0.6500	0.4106	0.045*	
C56	0.2024 (3)	0.3592 (3)	0.2663 (2)	0.0359 (9)	
H56	0.2230	0.2899	0.2297	0.043*	
C51	0.2972 (3)	0.4595 (3)	0.2953 (2)	0.0291 (8)	
C64	0.4464 (3)	0.1996 (4)	0.0155 (2)	0.0409 (10)	
C35	0.8778 (3)	0.7413 (3)	0.5012 (2)	0.0425 (10)	
H35	0.8450	0.7078	0.5480	0.051*	
C34	0.9876 (4)	0.8233 (4)	0.5127 (2)	0.0388 (9)	
C26	0.8040 (4)	0.4939 (3)	0.1368 (2)	0.0392 (9)	
H26	0.7130	0.4768	0.1352	0.047*	
C11	0.8064 (3)	0.8380 (3)	0.1951 (2)	0.0301 (8)	
C24	0.9996 (4)	0.4386 (4)	0.0929 (2)	0.0462 (10)	
C1	0.4294 (3)	0.8469 (3)	0.2931 (2)	0.0366 (9)	
C45	0.5071 (3)	0.2764 (3)	0.4681 (2)	0.0429 (10)	
H45	0.4593	0.2502	0.5125	0.051*	
C61	0.4559 (3)	0.3533 (3)	0.1713 (2)	0.0303 (8)	
C33	1.0383 (3)	0.8743 (3)	0.4477 (2)	0.0386 (9)	
H33	1.1156	0.9310	0.4581	0.046*	
C32	0.9748 (3)	0.8418 (3)	0.3661 (2)	0.0323 (9)	
H32	1.0081	0.8772	0.3203	0.039*	
C52	0.2652 (3)	0.5610 (3)	0.3473 (2)	0.0343 (9)	
H52	0.3283	0.6311	0.3652	0.041*	
C13	0.8696 (3)	0.9298 (4)	0.0745 (3)	0.0496 (11)	
H13	0.9018	0.9247	0.0192	0.059*	
C16	0.7705 (3)	0.9467 (3)	0.2344 (2)	0.0410 (10)	
H16	0.7349	0.9523	0.2886	0.049*	
C46	0.4587 (3)	0.3450 (3)	0.4141 (2)	0.0375 (9)	
H46	0.3760	0.3665	0.4221	0.045*	
C53	0.1411 (3)	0.5606 (3)	0.3732 (2)	0.0427 (10)	
H53	0.1192	0.6286	0.4104	0.051*	
C65	0.4357 (3)	0.3166 (3)	0.0160 (2)	0.0412 (10)	
H65	0.4251	0.3445	−0.0364	0.049*	
C54	0.0521 (3)	0.4603 (4)	0.3440 (3)	0.0456 (11)	
C62	0.4617 (3)	0.2325 (3)	0.1674 (2)	0.0410 (10)	
H62	0.4686	0.2027	0.2193	0.049*	
C12	0.8554 (3)	0.8315 (3)	0.1148 (2)	0.0376 (9)	
H12	0.8796	0.7579	0.0870	0.045*	
C3	0.2929 (3)	0.8243 (4)	0.1507 (3)	0.0510 (11)	
H3	0.2786	0.7777	0.0939	0.061*	
C25	0.8687 (4)	0.4115 (3)	0.0897 (2)	0.0474 (10)	
H25	0.8239	0.3382	0.0559	0.057*	
C42	0.6499 (3)	0.3546 (3)	0.3411 (2)	0.0402 (10)	
H42	0.7005	0.3835	0.2989	0.048*	
C7	0.3872 (4)	0.9522 (3)	0.3371 (3)	0.0502 (11)	
H7	0.4301	0.9835	0.3925	0.060*	
C15	0.7863 (4)	1.0465 (4)	0.1951 (3)	0.0550 (11)	
H15	0.7632	1.1209	0.2222	0.066*	
C5	0.2203 (4)	1.0022 (4)	0.2396 (4)	0.0676 (14)	
H5	0.1608	1.0566	0.2381	0.081*	
C31	0.8621 (3)	0.7572 (3)	0.3513 (2)	0.0298 (8)	
C66	0.4405 (3)	0.3943 (3)	0.0940 (2)	0.0366 (9)	
H66	0.4333	0.4767	0.0954	0.044*	
C22	1.0001 (3)	0.6237 (3)	0.1867 (2)	0.0355 (9)	
H22	1.0457	0.6967	0.2204	0.043*	
C14	0.8356 (4)	1.0353 (4)	0.1170 (3)	0.0567 (12)	
C55	0.0784 (3)	0.3600 (4)	0.2907 (2)	0.0420 (10)	
H55	0.0131	0.2923	0.2708	0.050*	
C6	0.2949 (4)	1.0184 (4)	0.3146 (3)	0.0626 (13)	
H6	0.2821	1.0845	0.3576	0.075*	
C43	0.7001 (3)	0.2859 (3)	0.3927 (2)	0.0448 (10)	
H43	0.7832	0.2651	0.3857	0.054*	
C4	0.2200 (4)	0.9173 (5)	0.1660 (3)	0.0639 (13)	
H4	0.1626	0.9225	0.1194	0.077*	
C63	0.4579 (3)	0.1534 (3)	0.0893 (3)	0.0455 (10)	
H63	0.4630	0.0703	0.0868	0.055*	
C23	1.0675 (4)	0.5431 (4)	0.1391 (2)	0.0415 (10)	
H23	1.1582	0.5604	0.1387	0.050*	
C2	0.3839 (3)	0.7860 (4)	0.2032 (2)	0.0383 (9)	

### Atomic displacement parameters (Å^2^)

**Table:**

	*U*^11^	*U*^22^	*U*^33^	*U*^12^	*U*^13^	*U*^23^
Cu	0.0353 (3)	0.0327 (3)	0.0323 (3)	0.0023 (2)	0.0083 (2)	0.0066 (2)
P2	0.0310 (5)	0.0316 (6)	0.0282 (5)	0.0027 (4)	0.0064 (4)	0.0066 (4)
P1	0.0349 (5)	0.0286 (6)	0.0290 (6)	0.0035 (4)	0.0091 (4)	0.0064 (4)
F2	0.0583 (14)	0.0789 (18)	0.0545 (15)	0.0237 (13)	0.0077 (12)	0.0347 (13)
F6	0.0701 (15)	0.0490 (15)	0.0420 (14)	0.0062 (12)	0.0095 (11)	−0.0088 (11)
F3	0.0638 (14)	0.0660 (16)	0.0371 (14)	0.0194 (12)	−0.0073 (11)	0.0010 (11)
F5	0.0332 (12)	0.0780 (19)	0.112 (2)	0.0122 (12)	0.0300 (13)	0.0290 (15)
F1	0.0968 (18)	0.0608 (17)	0.0609 (17)	0.0418 (15)	0.0314 (14)	0.0017 (13)
O1	0.0443 (15)	0.0395 (16)	0.0322 (15)	0.0081 (13)	0.0057 (12)	0.0018 (12)
O2	0.0529 (16)	0.0390 (16)	0.0324 (15)	0.0106 (13)	0.0041 (12)	0.0069 (12)
F4	0.143 (2)	0.0567 (18)	0.100 (2)	0.0193 (17)	0.0288 (19)	0.0494 (16)
C41	0.032 (2)	0.030 (2)	0.029 (2)	−0.0021 (16)	0.0061 (16)	0.0062 (16)
C21	0.039 (2)	0.031 (2)	0.030 (2)	0.0076 (18)	0.0099 (17)	0.0068 (17)
C44	0.041 (2)	0.044 (2)	0.034 (2)	0.0072 (19)	−0.0041 (19)	0.0148 (19)
C36	0.035 (2)	0.046 (3)	0.033 (2)	0.0048 (18)	0.0100 (18)	0.0094 (19)
C56	0.044 (2)	0.032 (2)	0.031 (2)	0.0059 (19)	0.0044 (18)	0.0056 (17)
C51	0.0278 (19)	0.035 (2)	0.029 (2)	0.0097 (17)	0.0056 (16)	0.0141 (17)
C64	0.042 (2)	0.044 (3)	0.032 (2)	0.004 (2)	0.0090 (18)	−0.005 (2)
C35	0.049 (2)	0.055 (3)	0.026 (2)	0.012 (2)	0.0094 (19)	0.0115 (19)
C34	0.042 (2)	0.048 (3)	0.026 (2)	0.017 (2)	−0.0014 (19)	−0.0015 (19)
C26	0.049 (2)	0.032 (2)	0.035 (2)	0.0017 (19)	0.0091 (19)	0.0065 (19)
C11	0.0290 (19)	0.028 (2)	0.032 (2)	0.0018 (16)	0.0037 (16)	0.0041 (17)
C24	0.073 (3)	0.039 (3)	0.034 (2)	0.026 (2)	0.022 (2)	0.008 (2)
C1	0.036 (2)	0.032 (2)	0.042 (3)	−0.0027 (18)	0.0118 (19)	0.0105 (19)
C45	0.043 (2)	0.060 (3)	0.030 (2)	0.011 (2)	0.0101 (18)	0.016 (2)
C61	0.0265 (18)	0.033 (2)	0.032 (2)	0.0024 (16)	0.0070 (16)	0.0078 (17)
C33	0.036 (2)	0.037 (2)	0.039 (3)	0.0058 (18)	0.0025 (19)	−0.0015 (19)
C32	0.038 (2)	0.030 (2)	0.030 (2)	0.0097 (18)	0.0093 (17)	0.0033 (17)
C52	0.029 (2)	0.036 (2)	0.039 (2)	0.0004 (17)	0.0070 (17)	0.0133 (18)
C13	0.060 (3)	0.051 (3)	0.047 (3)	0.010 (2)	0.016 (2)	0.026 (2)
C16	0.052 (2)	0.037 (3)	0.032 (2)	0.0044 (19)	0.0081 (18)	0.0023 (19)
C46	0.031 (2)	0.052 (3)	0.030 (2)	0.0082 (19)	0.0069 (17)	0.0060 (19)
C53	0.040 (2)	0.041 (3)	0.050 (3)	0.010 (2)	0.014 (2)	0.011 (2)
C65	0.059 (3)	0.033 (2)	0.030 (2)	0.003 (2)	0.0049 (19)	0.0053 (19)
C54	0.026 (2)	0.057 (3)	0.062 (3)	0.005 (2)	0.013 (2)	0.031 (2)
C62	0.058 (3)	0.031 (2)	0.038 (2)	0.012 (2)	0.0065 (19)	0.0129 (19)
C12	0.043 (2)	0.033 (2)	0.040 (2)	0.0082 (18)	0.0108 (18)	0.0107 (18)
C3	0.045 (2)	0.055 (3)	0.056 (3)	−0.001 (2)	−0.002 (2)	0.025 (2)
C25	0.074 (3)	0.022 (2)	0.043 (3)	0.009 (2)	0.010 (2)	−0.0016 (18)
C42	0.031 (2)	0.055 (3)	0.040 (2)	0.0080 (19)	0.0123 (18)	0.020 (2)
C7	0.057 (3)	0.037 (3)	0.055 (3)	0.005 (2)	0.018 (2)	0.005 (2)
C15	0.080 (3)	0.031 (3)	0.056 (3)	0.012 (2)	0.004 (3)	0.011 (2)
C5	0.051 (3)	0.051 (3)	0.116 (5)	0.016 (3)	0.033 (3)	0.043 (3)
C31	0.032 (2)	0.029 (2)	0.029 (2)	0.0068 (17)	0.0075 (16)	0.0047 (16)
C66	0.046 (2)	0.028 (2)	0.036 (2)	0.0039 (17)	0.0038 (18)	0.0086 (18)
C22	0.041 (2)	0.031 (2)	0.033 (2)	0.0043 (18)	0.0065 (18)	0.0028 (17)
C14	0.073 (3)	0.033 (3)	0.071 (3)	0.004 (2)	0.008 (3)	0.032 (2)
C55	0.031 (2)	0.045 (3)	0.049 (3)	−0.0023 (19)	−0.0003 (19)	0.014 (2)
C6	0.064 (3)	0.040 (3)	0.090 (4)	0.016 (2)	0.029 (3)	0.014 (3)
C43	0.033 (2)	0.058 (3)	0.051 (3)	0.015 (2)	0.011 (2)	0.023 (2)
C4	0.039 (3)	0.070 (4)	0.095 (4)	0.006 (3)	0.006 (3)	0.049 (3)
C63	0.061 (3)	0.028 (2)	0.046 (3)	0.005 (2)	0.008 (2)	0.003 (2)
C23	0.045 (2)	0.050 (3)	0.035 (2)	0.019 (2)	0.0181 (19)	0.012 (2)
C2	0.031 (2)	0.044 (3)	0.042 (3)	0.0002 (19)	0.0076 (18)	0.016 (2)

### Geometric parameters (Å, °)

**Table:**

Cu—O1	2.084 (2)	C45—H45	0.9500
Cu—O2	2.082 (2)	C61—C62	1.378 (4)
Cu—P1	2.2335 (15)	C61—C66	1.402 (4)
Cu—P2	2.2377 (10)	C33—C32	1.390 (4)
P2—C51	1.820 (3)	C33—H33	0.9500
P2—C61	1.826 (3)	C32—C31	1.396 (4)
P2—C41	1.827 (3)	C32—H32	0.9500
P1—C11	1.824 (3)	C52—C53	1.393 (4)
P1—C21	1.831 (3)	C52—H52	0.9500
P1—C31	1.835 (3)	C13—C14	1.373 (5)
F2—C44	1.366 (4)	C13—C12	1.381 (5)
F6—C64	1.364 (4)	C13—H13	0.9500
F3—C34	1.359 (4)	C16—C15	1.386 (5)
F5—C54	1.361 (4)	C16—H16	0.9500
F1—C24	1.364 (4)	C46—H46	0.9500
O1—C1	1.275 (4)	C53—C54	1.359 (5)
O2—C2	1.273 (4)	C53—H53	0.9500
F4—C14	1.366 (4)	C65—C66	1.378 (5)
C41—C46	1.386 (4)	C65—H65	0.9500
C41—C42	1.391 (4)	C54—C55	1.365 (5)
C21—C26	1.372 (5)	C62—C63	1.388 (5)
C21—C22	1.391 (4)	C62—H62	0.9500
C44—C43	1.366 (5)	C12—H12	0.9500
C44—C45	1.368 (5)	C3—C4	1.395 (6)
C36—C35	1.386 (5)	C3—C2	1.412 (5)
C36—C31	1.394 (4)	C3—H3	0.9500
C36—H36	0.9500	C25—H25	0.9500
C56—C55	1.385 (5)	C42—C43	1.373 (5)
C56—C51	1.393 (4)	C42—H42	0.9500
C56—H56	0.9500	C7—C6	1.392 (5)
C51—C52	1.389 (4)	C7—H7	0.9500
C64—C65	1.354 (5)	C15—C14	1.358 (5)
C64—C63	1.376 (5)	C15—H15	0.9500
C35—C34	1.359 (5)	C5—C6	1.364 (6)
C35—H35	0.9500	C5—C4	1.373 (6)
C34—C33	1.365 (5)	C5—H5	0.9500
C26—C25	1.376 (5)	C66—H66	0.9500
C26—H26	0.9500	C22—C23	1.384 (4)
C11—C12	1.391 (4)	C22—H22	0.9500
C11—C16	1.396 (5)	C55—H55	0.9500
C24—C23	1.359 (5)	C6—H6	0.9500
C24—C25	1.367 (5)	C43—H43	0.9500
C1—C7	1.418 (5)	C4—H4	0.9500
C1—C2	1.490 (5)	C63—H63	0.9500
C45—C46	1.391 (5)	C23—H23	0.9500
			
O2—Cu—O1	77.72 (10)	C14—C13—H13	121.1
O2—Cu—P1	117.65 (7)	C12—C13—H13	121.1
O1—Cu—P1	102.97 (7)	C15—C16—C11	120.7 (4)
O2—Cu—P2	100.68 (7)	C15—C16—H16	119.7
O1—Cu—P2	118.07 (7)	C11—C16—H16	119.7
P1—Cu—P2	128.82 (4)	C41—C46—C45	122.2 (3)
C51—P2—C61	103.42 (15)	C41—C46—H46	118.9
C51—P2—C41	102.55 (15)	C45—C46—H46	118.9
C61—P2—C41	101.30 (16)	C54—C53—C52	118.3 (3)
C51—P2—Cu	114.64 (12)	C54—C53—H53	120.8
C61—P2—Cu	111.83 (11)	C52—C53—H53	120.8
C41—P2—Cu	120.92 (11)	C64—C65—C66	118.6 (3)
C11—P1—C21	102.93 (16)	C64—C65—H65	120.7
C11—P1—C31	103.02 (15)	C66—C65—H65	120.7
C21—P1—C31	104.40 (15)	C53—C54—F5	118.3 (4)
C11—P1—Cu	113.85 (11)	C53—C54—C55	123.3 (3)
C21—P1—Cu	119.52 (12)	F5—C54—C55	118.4 (4)
C31—P1—Cu	111.40 (11)	C61—C62—C63	121.4 (3)
C1—O1—Cu	114.7 (2)	C61—C62—H62	119.3
C2—O2—Cu	115.2 (2)	C63—C62—H62	119.3
C46—C41—C42	117.5 (3)	C13—C12—C11	121.4 (3)
C46—C41—P2	124.2 (3)	C13—C12—H12	119.3
C42—C41—P2	118.3 (3)	C11—C12—H12	119.3
C26—C21—C22	117.9 (3)	C4—C3—C2	132.4 (4)
C26—C21—P1	119.2 (3)	C4—C3—H3	113.8
C22—C21—P1	122.9 (3)	C2—C3—H3	113.8
C43—C44—F2	118.1 (3)	C24—C25—C26	117.9 (4)
C43—C44—C45	123.7 (3)	C24—C25—H25	121.1
F2—C44—C45	118.1 (3)	C26—C25—H25	121.1
C35—C36—C31	120.8 (3)	C43—C42—C41	121.8 (3)
C35—C36—H36	119.6	C43—C42—H42	119.1
C31—C36—H36	119.6	C41—C42—H42	119.1
C55—C56—C51	120.2 (3)	C6—C7—C1	132.2 (4)
C55—C56—H56	119.9	C6—C7—H7	113.9
C51—C56—H56	119.9	C1—C7—H7	113.9
C52—C51—C56	119.2 (3)	C14—C15—C16	118.4 (4)
C52—C51—P2	117.3 (3)	C14—C15—H15	120.8
C56—C51—P2	123.5 (3)	C16—C15—H15	120.8
C65—C64—F6	119.8 (3)	C6—C5—C4	128.3 (4)
C65—C64—C63	123.2 (3)	C6—C5—H5	115.8
F6—C64—C63	116.9 (3)	C4—C5—H5	115.8
C34—C35—C36	118.5 (3)	C36—C31—C32	118.8 (3)
C34—C35—H35	120.8	C36—C31—P1	118.0 (3)
C36—C35—H35	120.8	C32—C31—P1	123.3 (3)
C35—C34—F3	118.3 (3)	C65—C66—C61	120.8 (3)
C35—C34—C33	123.0 (3)	C65—C66—H66	119.6
F3—C34—C33	118.8 (3)	C61—C66—H66	119.6
C21—C26—C25	121.9 (4)	C23—C22—C21	121.4 (3)
C21—C26—H26	119.0	C23—C22—H22	119.3
C25—C26—H26	119.0	C21—C22—H22	119.3
C12—C11—C16	118.4 (3)	C15—C14—F4	119.4 (4)
C12—C11—P1	122.3 (3)	C15—C14—C13	123.4 (4)
C16—C11—P1	119.1 (3)	F4—C14—C13	117.2 (4)
C23—C24—F1	118.0 (4)	C54—C55—C56	118.6 (3)
C23—C24—C25	123.1 (4)	C54—C55—H55	120.7
F1—C24—C25	118.9 (4)	C56—C55—H55	120.7
O1—C1—C7	119.4 (4)	C5—C6—C7	129.3 (4)
O1—C1—C2	116.5 (3)	C5—C6—H6	115.3
C7—C1—C2	124.2 (4)	C7—C6—H6	115.3
C44—C45—C46	116.8 (3)	C44—C43—C42	117.9 (3)
C44—C45—H45	121.6	C44—C43—H43	121.0
C46—C45—H45	121.6	C42—C43—H43	121.0
C62—C61—C66	118.3 (3)	C5—C4—C3	128.5 (4)
C62—C61—P2	124.3 (3)	C5—C4—H4	115.7
C66—C61—P2	117.3 (3)	C3—C4—H4	115.7
C34—C33—C32	118.9 (3)	C64—C63—C62	117.6 (4)
C34—C33—H33	120.6	C64—C63—H63	121.2
C32—C33—H33	120.6	C62—C63—H63	121.2
C33—C32—C31	120.1 (3)	C24—C23—C22	117.8 (3)
C33—C32—H32	120.0	C24—C23—H23	121.1
C31—C32—H32	120.0	C22—C23—H23	121.1
C51—C52—C53	120.3 (3)	O2—C2—C3	119.2 (4)
C51—C52—H52	119.8	O2—C2—C1	115.9 (3)
C53—C52—H52	119.8	C3—C2—C1	124.9 (4)
C14—C13—C12	117.8 (4)		
			
O2—Cu—P2—C51	−49.50 (14)	C56—C51—C52—C53	2.8 (5)
O1—Cu—P2—C51	32.18 (15)	P2—C51—C52—C53	−175.6 (3)
P1—Cu—P2—C51	171.23 (12)	C12—C11—C16—C15	−1.5 (5)
O2—Cu—P2—C61	67.82 (13)	P1—C11—C16—C15	−175.7 (3)
O1—Cu—P2—C61	149.49 (14)	C42—C41—C46—C45	−2.7 (5)
P1—Cu—P2—C61	−71.45 (12)	P2—C41—C46—C45	175.6 (3)
O2—Cu—P2—C41	−173.11 (14)	C44—C45—C46—C41	0.4 (5)
O1—Cu—P2—C41	−91.43 (16)	C51—C52—C53—C54	−2.2 (5)
P1—Cu—P2—C41	47.62 (14)	F6—C64—C65—C66	−179.5 (3)
O2—Cu—P1—C11	23.73 (15)	C63—C64—C65—C66	2.1 (6)
O1—Cu—P1—C11	−59.05 (14)	C52—C53—C54—F5	−179.4 (3)
P2—Cu—P1—C11	157.35 (12)	C52—C53—C54—C55	0.2 (6)
O2—Cu—P1—C21	−98.37 (15)	C66—C61—C62—C63	2.6 (5)
O1—Cu—P1—C21	178.85 (14)	P2—C61—C62—C63	−178.4 (3)
P2—Cu—P1—C21	35.25 (15)	C14—C13—C12—C11	1.0 (6)
O2—Cu—P1—C31	139.70 (14)	C16—C11—C12—C13	0.5 (5)
O1—Cu—P1—C31	56.92 (14)	P1—C11—C12—C13	174.5 (3)
P2—Cu—P1—C31	−86.68 (12)	C23—C24—C25—C26	−1.2 (6)
O2—Cu—O1—C1	−0.8 (2)	F1—C24—C25—C26	179.0 (3)
P1—Cu—O1—C1	115.1 (2)	C21—C26—C25—C24	−0.2 (6)
P2—Cu—O1—C1	−96.5 (2)	C46—C41—C42—C43	3.5 (5)
O1—Cu—O2—C2	−0.3 (2)	P2—C41—C42—C43	−175.0 (3)
P1—Cu—O2—C2	−98.7 (2)	O1—C1—C7—C6	−174.7 (4)
P2—Cu—O2—C2	116.4 (2)	C2—C1—C7—C6	4.5 (6)
C51—P2—C41—C46	−7.0 (3)	C11—C16—C15—C14	1.0 (6)
C61—P2—C41—C46	−113.7 (3)	C35—C36—C31—C32	0.0 (5)
Cu—P2—C41—C46	122.1 (3)	C35—C36—C31—P1	179.3 (3)
C51—P2—C41—C42	171.3 (3)	C33—C32—C31—C36	0.6 (5)
C61—P2—C41—C42	64.6 (3)	C33—C32—C31—P1	−178.6 (2)
Cu—P2—C41—C42	−59.5 (3)	C11—P1—C31—C36	150.2 (3)
C11—P1—C21—C26	−115.4 (3)	C21—P1—C31—C36	−102.5 (3)
C31—P1—C21—C26	137.3 (3)	Cu—P1—C31—C36	27.8 (3)
Cu—P1—C21—C26	12.0 (3)	C11—P1—C31—C32	−30.5 (3)
C11—P1—C21—C22	62.7 (3)	C21—P1—C31—C32	76.8 (3)
C31—P1—C21—C22	−44.6 (3)	Cu—P1—C31—C32	−152.9 (2)
Cu—P1—C21—C22	−169.9 (2)	C64—C65—C66—C61	−0.1 (5)
C55—C56—C51—C52	−1.4 (5)	C62—C61—C66—C65	−2.1 (5)
C55—C56—C51—P2	176.9 (3)	P2—C61—C66—C65	178.8 (3)
C61—P2—C51—C52	−156.6 (3)	C26—C21—C22—C23	0.0 (5)
C41—P2—C51—C52	98.3 (3)	P1—C21—C22—C23	−178.1 (3)
Cu—P2—C51—C52	−34.6 (3)	C16—C15—C14—F4	178.7 (4)
C61—P2—C51—C56	25.0 (3)	C16—C15—C14—C13	0.6 (7)
C41—P2—C51—C56	−80.0 (3)	C12—C13—C14—C15	−1.6 (6)
Cu—P2—C51—C56	147.0 (2)	C12—C13—C14—F4	−179.7 (3)
C31—C36—C35—C34	−0.4 (5)	C53—C54—C55—C56	1.2 (6)
C36—C35—C34—F3	178.8 (3)	F5—C54—C55—C56	−179.2 (3)
C36—C35—C34—C33	0.3 (6)	C51—C56—C55—C54	−0.6 (5)
C22—C21—C26—C25	0.7 (5)	C4—C5—C6—C7	−1.9 (8)
P1—C21—C26—C25	178.9 (3)	C1—C7—C6—C5	−3.0 (7)
C21—P1—C11—C12	18.1 (3)	F2—C44—C43—C42	179.2 (3)
C31—P1—C11—C12	126.5 (3)	C45—C44—C43—C42	−0.7 (6)
Cu—P1—C11—C12	−112.7 (3)	C41—C42—C43—C44	−1.8 (6)
C21—P1—C11—C16	−167.9 (3)	C6—C5—C4—C3	2.3 (8)
C31—P1—C11—C16	−59.5 (3)	C2—C3—C4—C5	1.9 (7)
Cu—P1—C11—C16	61.3 (3)	C65—C64—C63—C62	−1.6 (6)
Cu—O1—C1—C7	−179.0 (2)	F6—C64—C63—C62	179.9 (3)
Cu—O1—C1—C2	1.7 (4)	C61—C62—C63—C64	−0.8 (5)
C43—C44—C45—C46	1.3 (6)	F1—C24—C23—C22	−178.3 (3)
F2—C44—C45—C46	−178.5 (3)	C25—C24—C23—C22	1.9 (6)
C51—P2—C61—C62	−90.2 (3)	C21—C22—C23—C24	−1.3 (5)
C41—P2—C61—C62	15.8 (3)	Cu—O2—C2—C3	−179.5 (2)
Cu—P2—C61—C62	145.9 (3)	Cu—O2—C2—C1	1.3 (4)
C51—P2—C61—C66	88.9 (3)	C4—C3—C2—O2	177.4 (4)
C41—P2—C61—C66	−165.1 (2)	C4—C3—C2—C1	−3.4 (6)
Cu—P2—C61—C66	−35.0 (3)	O1—C1—C2—O2	−2.1 (5)
C35—C34—C33—C32	0.3 (5)	C7—C1—C2—O2	178.7 (3)
F3—C34—C33—C32	−178.2 (3)	O1—C1—C2—C3	178.7 (3)
C34—C33—C32—C31	−0.8 (5)	C7—C1—C2—C3	−0.5 (6)
